# Physical fitness is predictive for 5‐year survival in older adults with intellectual disabilities

**DOI:** 10.1111/jar.12589

**Published:** 2019-04-05

**Authors:** Alyt Oppewal, Thessa I.M. Hilgenkamp

**Affiliations:** ^1^ Department of General Practice, Intellectual Disability Medicine Erasmus MC, University Medical Center Rotterdam Rotterdam The Netherlands; ^2^ Department of Kinesiology and Nutrition University of Illinois Chicago Illinois

**Keywords:** activity, intellectual disabilities, mortality, older adults, physical capacity

## Abstract

**Background:**

The very low physical fitness levels of people with intellectual disabilities (ID) may influence their life expectancy. Therefore, we investigated the predictive value of physical fitness for survival in older adults with intellectual disabilities.

**Method:**

In the Healthy Ageing and Intellectual Disabilities (HA‐ID) study,the physical fitness levels of 900 older adults (≥50 years; 61.5 ± 8.1 years) were measured at baseline. All‐cause mortality was collected over a 5‐year follow‐up period. Cox proportional hazard models were used to determine the association between each physical fitness test and survival, adjusted for age, sex, level of ID, and Down syndrome.

**Results:**

The physical fitness components that were independently predictive for survival were manual dexterity (HR = 0.96 [0.94–0.98]), visual reaction time (HR = 1.57 [1.28–1.94]), balance (HR = 0.97 [0.95–0.99]), comfortable gait speed (HR = 0.65 [0.54–0.78]), fast gait speed (HR = 0.81 [0.72–0.91]), grip strength (HR = 0.97 [0.94–0.99]) and cardiorespiratory fitness (HR = 0.997 [0.995–0.999]), with a better physical fitness showing a lower mortality risk.

**Conclusion:**

We showed for the first time that physical fitness was independently associated with survival in older adults with intellectual disabilities. Improving and maintaining physical fitness must become an essential part of care and support for this population.

## 1 INTRODUCTION

The life expectancy of people with intellectual disabilities has increased due to improved care (Coppus, 2013). However, these additional years are not necessarily spent in good health and with good quality of life (Coppus, 2013). People with intellectual disabilities often have more health problems earlier in life (Reppermund & Trollor, 2016), and older adults with intellectual disabilities are more frail at a younger age than older adults in the general population (Schoufour, Mitnitski, Rockwood, Evenhuis, & Echteld, 2013). Physical fitness is an important factor with regard to these health problems and frailty (American College of Sports Medicine, 2018; Bernabei et al., 2014; Bouchard & Shephard, 1994; Rodriguez‐Manas et al., 2013), and may also be an important factor for increasing survival in people with intellectual disabilities. Physical fitness may especially be an important area to focus on because very low physical fitness levels have been found in people with intellectual disabilities (Golubovic, Maksimovic, Golubovic, & Glumbic, 2012; Hilgenkamp, van Wijck, & Evenhuis, 2012b; Lahtinen, Rintala, & Malin, 2007; Oppewal, Hilgenkamp, van Wijck, & Evenhuis, 2013; Salaun & Berthouze‐Aranda, 2012). Fitness levels of older adults with intellectual disabilities aged 50 years and over have been found to be comparable to or even worse than those of adults in the general population aged 20 years older (Hilgenkamp et al., 2012b). These low physical fitness levels may have a negative impact on survival, and on their health and quality of life at older age.

In the general population, physical fitness has indeed been found to be related to survival (American College of Sports Medicine, 2018; Clegg, Young, Iliffe, Rikkert, & Rockwood, 2013; U.S. Department of Health & Human Services, 2008). For example, low cardiorespiratory fitness is a major independent risk factor for all‐cause mortality (Lee, Artero, Sui, & Blair, 2010; U.S. Department of Health & Human Services, 2008). Additionally, low grip strength, slow gait speed and worse performance on sit‐to‐stands have also been found to be associated with an increased risk of premature mortality in the general older population (most studies included adults over 55 years of age; Bohannon, 2008; Cooper, Kuh, & Hardy, 2010; Elbaz et al., 2013; Studenski et al., 2011). Results from studies performed in the general population cannot be generalized to people with intellectual disabilities, because the predictive value of physical fitness for survival may be different in people with intellectual disabilities. Many of them already have multiple disabilities and/or chronic diseases at a younger age (Reppermund & Trollor, 2016). This may influence the impact of low physical fitness levels on survival. Also people with intellectual disabilities have low physical fitness levels across the lifespan (Golubovic et al., 2012; Hilgenkamp et al., 2012b; Lahtinen et al., 2007; Oppewal et al., 2013; Salaun & Berthouze‐Aranda, 2012). Therefore, the age‐related decline in physical fitness and associated risk of negative health outcomes and premature mortality may be less pronounced in older adults with intellectual disabilities than in the general older population.

Previously, we did find that low physical fitness was predictive for a decline in the ability to perform basic and instrumental activities of daily living and for a decline in mobility, over a period of three years (Oppewal, Hilgenkamp, van Wijck, Schoufour, & Evenhuis, 2014, 2015). Even more important, physical fitness may be an important target area to increase survival in this population and to help them age healthier. It is therefore critical to understand the relationship between physical fitness and survival in this population, as this has not been studied yet. Obtaining insight into the importance of being physically fit into older age may help improve the life expectancy of people with intellectual disabilities, and the quality of life of those additional years. Therefore, the aim of this study was to investigate the predictive value of physical fitness for survival in older adults with intellectual disabilities. Secondary, we will assess the accuracy of the different physical fitness components in predicting survival in older adults with intellectual disabilities.

## 2 METHODS

### 2.1 Study design and participants

This study is part of the Healthy Ageing and Intellectual Disabilities (HA‐ID) study, a prospective cohort study performed by three ID care organizations and the Chair of Intellectual Disability Medicine at the Erasmus MC, University Medical Center Rotterdam in the Netherlands. The HA‐ID study study focused on the health of older adults with intellectual disabilities (≥50 years), and started off in 2008 with investigating physical activity and fitness, nutrition and nutritional state, and mood and anxiety. At the start of the study from November 2008 to July 2010, all 2,322 clients aged 50 years and over receiving care and support of the participating care organizations were invited to participate, without applying any exclusion criteria. All participants or their legal representatives provided informed consent for participation, resulting in a near‐representative sample of 1,050 participants. Adults without any form of registered care or support were not included, and adults who only visit a day care centre or only receive ambulatory care were underrepresented, as well as adults aged 80–84 years old. Females were slightly overrepresented. More details about the study design, recruitment and representativeness of the study sample are described elsewhere (Hilgenkamp et al., 2011). Of the total 1,050 participants, 900 participated in the physical fitness assessment. Feasibility of the physical fitness tests differed per subgroup, and participants with severe and profound intellectual disabilities and wheelchair users had the most trouble performing the tests (Hilgenkamp, van Wijck, & Evenhuis, 2013). Therefore, adults with severe and profound intellectual disabilities and wheelchair users were underrepresented in the physical fitness assessments when compared to the total HA‐ID study sample (with chi‐square tests). Adults aged 50–59 years, adults with borderline and mild intellectual disabilities, and those who walk independently were overrepresented (dropout described in more detail elsewhere) (Hilgenkamp et al., 2012b). Baseline data collection was conducted between November 2008 and July 2010. Follow‐up data on all‐cause mortality were collected during a 5‐year follow‐up period from the baseline measurements up to March 2015.

This study adheres to the guidelines of the Declaration of Helsinki (Helsinki, 2013) and was approved by the Medical Ethics Committee of the Erasmus MC, University Medical Center Rotterdam (MEC 2008‐234 and MEC 2011‐309).

### 2.2 Measurements

#### 2.2.1 Personal characteristics

Baseline data on age and sex were collected from the administrative electronic systems. Level of intellectual disabilities was collected from psychologists' and behavioural therapists' files, categorized as borderline (intelligence quotient [IQ] = 70–80), mild (IQ = 55–70), moderate (IQ = 35–55), severe (IQ = 25–35) or profound (IQ < 25). Genetic syndrome causing the intellectual disability was retrieved from the medical files. Because Down syndrome was the only genetic syndrome that was present in a substantial part of the study sample (14.1% vs. <1% for other genetic syndromes), we only included this syndrome as a subgroup in this study.

#### 2.2.2 Physical fitness

Physical fitness was measured at baseline during a physical fitness assessment at locations close or familiar to participants. Physiotherapists, occupational therapists and physical activity instructors with vast experience in working with people with intellectual disabilities conducted the tests. Prior to data collection, they all received an instruction manual and a 2‐day training for the execution of the tests, in which they practised the assessment of the tests. During the training test, results were compared between different test instructors to assure similarity of the results. Also, it was emphasized that results should only be recorded if the test instructor was convinced that participants understood the task and performed with maximal effort. The physical fitness tests used are described in detail elsewhere (Oppewal et al., 2014). In short, we measured manual dexterity with the Box and Block test (BBT; Mathiowetz, Volland, Kashman, & Weber, 1985), reaction time with an auditive (RTA) and a visual (RTV) reaction time task (Berg, 1989; Dunn, 1978), balance with the Berg Balance Scale (BBS; Berg, 1989; Berg, Wood‐Dauphinee, Williams, & Maki, 1992), gait speed while walking at comfortable speed (GSC) and while walking at fast speed (GSF; Bohannon, 1997), grip strength (GS) with a Jamar Hand Dynamometer (Fess & Moran, 1981), muscular endurance with the 30‐s Chair stand (30s CS; Rikli & Jones, 2001), flexibility with the extended version of the modified back saver sit and reach test (EMBSSR; Hilgenkamp, van Wijck, & Evenhuis, 2010; Hui & Yuen, 2000) and finally cardiorespiratory fitness with the 10‐m incremental shuttle walking test (ISWT; Singh, Morgan, Scott, Walters, & Hardman, 1992). Feasibility and reliability of these instruments were good in older adults with intellectual disabilities (Hilgenkamp, van Wijck, & Evenhuis, 2012a; Hilgenkamp et al., 2013), and validity and reliability have also been confirmed in the general population.

#### 2.2.3 All‐cause mortality

All‐cause mortality data were collected after a 5‐year follow‐up period. The client administration departments of the care organizations identified deceased participants and the time of death. Additionally, they checked whether all remaining participants were still registered at the care organizations. If not, they provided us with the date of deregistration.

### 2.3 Statistical analyses

Descriptive statistics are provided for the total study sample, consisting of participants who had performed at least one physical fitness test, and the study participants who survived, were deceased or deregistered at the time of the 5‐year follow‐up. To assess whether loss to follow‐up was selective, differences in participant characteristics were analysed between the deceased and deregistered participants and those who survived with independent *t* tests for continuous variables and chi‐square tests for categorical variables. Additionally, differences in physical fitness between those who survived and the deceased were analysed with independent *t* tests.

The relationship between the physical fitness components and survival was assessed with survival analyses, with log‐rank tests and Cox proportional hazard models. Data on participants lost to follow‐up were censored on the date of death, deregistration or at the end of the study, whichever one came first. To assess the proportional hazards assumption, we used the scaled Schoenfeld residuals and plotted *β*(*t*) for the variables against time. To evaluate the risk of informative censoring, characteristics of those lost to follow‐up were analysed previously (Schoufour, Mitnitski, Rockwood, Evenhuis, & Echteld, 2015). The assumptions of proportional hazards and non‐informative censoring were sufficiently met.

Log‐rank tests were used to assess differences in survival between groups differing in age, sex, level of intellectual disabilities and Down syndrome. Cox proportional hazard models were used to assess the predictive value of each physical fitness component for survival. Additionally, a Cox proportional hazard model adjusted for age, sex, level of intellectual disabilities and Down syndrome was calculated. To allow for a better interpretation of the hazard ratios, the units of auditive and visual reaction time were divided by 10 (ms to cs). We checked for multicollinearity with the variance inflation factor (VIF), which had to be below 10. The C‐statistic was calculated for all Cox proportional hazard models of the physical fitness components that were significantly related to survival, to allow for a formal comparison of the prognostic value of the different models. The higher the C‐statistic, the better the model discriminates between participants who survived and those who died, based on time‐to‐event data. The C‐statistic was calculated for the subset of the data with participants that had complete data on the physical fitness components that were significantly related to survival.

Additionally, to estimate the accuracy of the physical fitness tests in predicting survival, we constructed receiver operator characteristic (ROC) curves based on the saved probabilities from logistic regression models, with survival as the dependent variable (as a binary outcome) and each physical fitness component, age, sex, level of intellectual disabilities and Down syndrome as independent variables. We calculated the area under the curve (AUC), which determines the ability of the test to discriminate between the survived and deceased participants. A value of 1 means that the test perfectly discriminates between the survived and deceased, a value of 0.5 means that there is a 50‐50 probability the test discriminates correctly. An AUC of >0.9 is often categorized as high accuracy, 0.7–0.9 as moderate accuracy and useful for some purposes, and 0.5–0.7 as low accuracy (Swets, 1988).

Analyses were performed with the Statistical Package for Social Sciences (SPSS) version 21 (IBM Corporation, New York) and R version 3.4.3 (R Foundation, Core Team, Vienna, Austria). Statistical significance was set at *p* < 0.05.

## 3 RESULTS

### 3.1 Baseline personal characteristics of the study sample

Table 1 presents the personal characteristics for the total study sample, and separately for the survived, deceased, and deregistered participants. At baseline, the mean age was 61.5 ± 8.1 years, and 49.1% was female. Of the 900 participants taking part in the physical fitness assessment at baseline, 172 (19.1%) died, and 50 (5.6%) were deregistered during the follow‐up period (4.7 ± 1.4 years, 0–6.3 years). Deregistered participants were significantly younger than the deceased (*t* = 5.3, *p *< 0.001). Participants who died were significantly older than the participants who survived (*t* = 6.0, *p *< 0.001) and had more often Down syndrome (χ^2^ = 16.9, *p *< 0.001). Participants were deregistered because of deteriorated health (*n = *7), other non‐medical reasons (*n = *11) and unknown reasons (*n = *32).

**Table 1:** Baseline personal characteristics of the study population

	Total *N* = 900 (100%)	Survived *n* = 678 (75.3%)	Deceased *n* = 172 (19.1%)	Deregistered *n* = 50 (5.6%)
Age^a^ (*M* ± *SD*) *n* (% of row)	61.5 ± 8.1	60.7 ± 7.4**	65.4 ± 9.7	59.5 ± 5.9*
50–59 yr	429 (100%)	349 (81.4%)	54 (12.6%)	26 (6.1%)
60–69 yr	310 (100%)	225 (72.6%)	63 (20.3%)	22 (7.1%)
70–79 yr	141 (100%)	97 (68.8%)	42 (29.8%)	2 (1.4%)
80 + yr	20 (100%)	7 (35.0%)	13 (65.0%)	0
Sex *n* (% of row)				
Female	442 (100%)	334 (75.6%)	78 (17.6%)	30 (6.8%)
Male	458 (100%)	344 (75.1%)	94 (20.5%)	20 (4.4%)
Level of intellectual disabilities *n* (% of row)				
Borderline	30 (100%)	26 (86.7%)	2 (6.7%)	2 (6.7%)
Mild	200 (100%)	156 (78.0%)	34 (17.0%)	10 (5.0%)
Moderate	441 (100%)	326 (73.9%)	89 (20.2%)	26 (5.9%)
Severe	140 (100%)	109 (77.9%)	24 (17.1%)	7 (5.0%)
Profound	68 (100%)	47 (69.1%)	18 (26.5%)	3 (4.4%)
Unknown	21 (100%)	14 (66.7%)	5 (23.8%)	2 (9.5%)
Down syndrome *n* (% of row)				
No	618 (100%)	487 (78.8%)**	106 (17.2%)	25 (4.0%)
Yes	127 (100%)	76 (59.8%)	40 (31.5%)	11 (8.7%)
Unknown	155 (100%)	115 (74.2%)	26 (16.8%)	14 (9.0%)

Notes

M: mean; *SD*: standard deviation; *n*: number of participants.

a Age at time of inclusion in study.

* Indicating a significant difference between deregistered and deceased participants, *p *< 0.05.

** Indicating a significant difference between survived and deceased participants, *p *< 0.05.

### 3.2 Baseline physical fitness

Table 2 presents the physical fitness results for the total study sample, and separately for the survived and the deceased. Participants who died had a significantly worse manual dexterity (*t *= −4.1, *p *< 0.001), auditive (*t = *2.4, *p *= 0.020) and visual reaction time (*t = *2.6, *p *= 0.010), balance (*t *= −2.3, *p *= 0.023), comfortable (*t *= −5.7, *p *< 0.001) and fast walking speed (*t *= −4.0, *p* < 0.001), grip strength (*t *= −2.9, *p* = 0.004), and cardiorespiratory fitness (*t *= −3.8, *p <* 0.001) than those who survived.

**Table 2:** Physical fitness results of the study population

	Total *N* = 900 (100%)	Survived *n* = 678 (75.3%)	Deceased *n* = 172 (19.1%)
Physical fitness (*M* ± *SD*)			
Manual dexterity *n = *743, in no. of blocks	28.7 ± 12.7	29.5 ± 12.4*	24.4 ± 13.0
Auditive reaction time *n = *566, in ms	1,044.0 ± 1,019.4	991.8 ± 983.4*	1,314.8 ± 1,230.2
Visual reaction time *n = *556, in ms	1,074.3 ± 849.4	1,015.6 ± 744.1*	1,362.4 ± 1,164.6
Balance *n = *508, points out of 56	47.2 ± 9.8	47.7 ± 9.1*	43.6 ± 12.9
Comfortable gait speed *n = *710, in km/h	3.5 ± 1.3	3.6 ± 1.2*	2.8 ± 1.21
Fast gait speed *n = *557, in km/h	6.5 ± 3.1	6.86 ± 3.1*	5.1 ± 2.8
Grip strength *n = *725, in kg	24.6 ± 10.0	25.1 ± 9.9*	22.2 ± 10.3
Muscular endurance *n = *528, in no. of reps	9.4 ± 3.3	9.5 ± 3.3	9.0 ± 3.3
Flexibility *n = *634, cm	−5.5 ± 14.1	−5.1 ± 13.9	−7.1 ± 14.2
Cardiorespiratory fitness *n = *590, in m	243.1 ± 173.0	253.3 ± 173.3*	173.0 ± 149.7

Notes

M: mean; *n*: number of participants; no. of blocks: number of blocks; no. of reps: number of repetitions; *SD*: standard deviation.

* Indicating a significant difference between survived and deceased participants, *p* < 0.05.

### 3.3 Five‐year survival

The log‐rank test showed significant differences in survival by age (χ^2^ = 65.6, *p* < 0.001) and Down syndrome (χ^2^ = 17.0, *p* < 0.001). Older people and people with Down syndrome were more likely to die in 5 years of follow‐up. No significant differences were found for sex and level of intellectual disabilities.

### 3.4 Physical fitness and survival

Physical fitness components that were significantly related to survival in the univariate Cox proportional hazard models were manual dexterity, auditive and visual reaction time, balance, comfortable and fast gait speed, grip strength, and cardiorespiratory fitness (model 1, Table 3). Except for auditive reaction time, all of these fitness components remained significant predictors for survival after adjusting for age, sex, level of intellectual disabilities and Down syndrome (model 2, Table 3). For visual reaction time, each unit (1 cs) decrease resulted in 0.5% lower mortality risk, for manual dexterity each unit (no. of blocks) increase resulted in a 4% lower mortality risk, for balance (points) this was 3%, for comfortable gait speed (km/h) 35%, for fast gait speed (km/h) 19%, for grip strength (kg) 3%, and for cardiorespiratory fitness (m) 0.3%. The C‐statistic of the models with the physical fitness components that were significantly associated with survival did not differ significantly from each other, meaning that all models were equally good at discriminating between participants who survived and those who died.

**Table 3:** Hazard ratios for 5‐year all‐cause mortality for physical fitness

Physical fitness	Model 1			Model 2			Model χ^2^	C‐statistic
	B (SE)	*HR *(95% *CI*)	Wald	B (SE)	*HR *(95% *CI*)	Wald		
Manual dexterity (no. of blocks)^a^	−0.03 (0.01)	0.97 (0.95–0.98)	18.1**	−0.04 (0.01)	0.96 (0.94–0.98)	15.3**	81.6**	0.64 (0.49–0.78)
Auditive reaction time (cs)^b^	0.002 (0.001)	1.002 (1.000–1.003)	7.1**	0.001 (0.001)^c^	1.001 (1.000–1.003)	2.2	60.0**	‐
Visual reaction time (cs)^b^	0.003 (0.001)	1.003 (1.001–1.005)	12.5**	0.005 (0.001)^c^	1.005 (1.002–1.007)	18.1**	57.9**	0.65 (0.50–0.79)
Balance (points out of 56)^a^	−0.03 (0.01)	0.97 (0.95–0.99)	9.3**	−0.03 (0.01)	0.97 (0.95–0.99)	8.3**	25.1**	0.66 (0.51–0.81)
Comfortable gait speed (km/h)^a^	−0.48 (0.09)	0.62 (0.53–0.73)	31.4**	−0.43 (0.09)	0.65 (0.54–0.78)	21.2**	65.9**	0.68 (0.56–0.80)
Fast gait speed (km/h)^a^	−0.20 (0.05)	0.82 (0.74–0.90)	15.2**	−0.21 (0.06)	0.81 (0.72–0.91)	13.7**	31.8**	0.66 (0.53–0.79)
Grip strength (kg)^a^	−0.03 (0.01)	0.97 (0.95–0.99)	8.0**	−0.03 (0.01)	0.97 (0.94–0.99)	7.2**	71.8**	0.63 (0.48–0.78)
Muscular endurance (no of reps)^a^	−0.04 (0.05)	0.96 (0.88–1.05)	0.9	−0.04 (0.05)	0.96 (0.87–1.05)	0.8	18.9**	–
Flexibility (cm)^a^	−0.01 (0.01)	0.99 (0.98–1.00)	1.8	−0.01 (0.01)	0.99 (0.98–1.01)	0.5	37.8**	–
Cardiorespiratory fitness (m)^a^	−0.003 (0.001)	0.997 (0.995–0.998)	14.0**	−0.003 (0.001)	0.997 (0.995–0.999)	11.1**	35.9**	0.66 (0.51–0.81)

Notes

Model 1 univariate cox proportional hazard model. Model 2 multivariate cox proportional hazard model, adjusted for age, sex, level of intellectual disabilities and Down syndrome. B: beta coefficient; CI: confidence interval; HR: hazard ratio; SE: standard error; Wald: Wald statistic; χ^2^: chi‐square.

a A higher score represents a better performance.

b A lower score represents a better performance.

c Units of auditive and visual reaction time in cs to allow for better interpretation of the HR.

* *p* < 0.05.

** *p* < 0.01.

Table 4 presents the AUCs based on the logistic regression models. Manual dexterity (AUC = 0.71), comfortable gait speed (AUC = 0.72), fast gait speed (AUC = 0.70) and cardiorespiratory fitness (AUC = 0.70) had a moderate accuracy in discriminating between the survived and deceased participants. Balance (AUC = 0.67), grip strength (AUC = 0.69) and visual reaction time (AUC = 0.69) had AUCs just under 0.7. The AUCs provided similar results as the C‐statistic based on the Cox models.

**Table 4:** Areas under the curve of the physical fitness tests with regard to discriminating between the participants who survived and those who died

Physical fitness tests	AUC
Manual dexterity	0.71
Visual reaction time	0.69
Balance	0.67
Comfortable gait speed	0.72
Fast gait speed	0.70
Grip strength	0.69
Cardiorespiratory fitness	0.70

Note

AUC: Area under the curve.

## 4 DISCUSSION

This is the first study to assess whether physical fitness is predictive for survival in older adults with intellectual disabilities, over a 5‐year follow‐up period. Over this follow‐up period, 172 out of 900 participants (19.1%) died. The physical fitness components that were predictive for survival were manual dexterity, visual reaction time, balance, comfortable and fast gait speed, grip strength, and cardiorespiratory fitness, with a better physical fitness showing a lower mortality risk. These results stress the need for being physically fit into older age to increase survival.

As seen in studies in the general population, we found physical fitness to be related to survival in older adults with intellectual disabilities (Bohannon, 2008; Cooper et al., 2010; Elbaz et al., 2013; Lee et al., 2010; Studenski et al., 2011; U.S. Department of Health & Human Services, 2008). There may be several explanations for this relationship. Physical fitness performance places demands on multiple organ systems such as heart, lungs, circulatory, musculoskeletal and nervous systems. Impairments in physical fitness may be a reflection of impairments in these systems, and therefore, physical fitness may reflect one's health status. However, in the general population, this relationship is also seen in studies with younger and healthy participants, and in studies excluding people with health problems (Cooper et al., 2010). Another pathway may be through the normal ageing process. Ageing in itself results in poorer physical fitness, for example, the well‐known age‐related decline in muscle mass and strength, called sarcopenia (Cruz‐Jentoft et al., 2010). With low physical fitness levels, one may be more prone to negative health conditions. For example, both cardiorespiratory fitness and muscle mass and function have been found to be related to cardiovascular risk factors such as insulin sensitivity, blood lipid profile and blood pressure (American College of Sports Medicine, 2018; Lee et al., 2010; Strasser & Pesta, 2013). A poor cardiovascular risk profile increases the risk of cardiovascular diseases and thereby mortality risk (Yang et al., 2012). Additionally, physical fitness is an important marker of frailty (Bernabei et al., 2014; Rodriguez‐Manas et al., 2013), which in turn is also related to survival (Clegg et al., 2013; Schoufour et al., 2015).

The accuracy of the physical fitness components in discriminating successfully between the survived and deceased participants was low to moderate, which may hamper the suitability as a purely discriminative clinical test. One possible explanation for this is that the physical fitness levels of our study sample were already very low. Baseline physical fitness levels were comparable or worse to those of older adults in the general population aged 20–30 years older. (Hilgenkamp et al., 2012b) The higher spectrum of physical fitness scores in our sample is missing, which limits the range of fitness scores, thereby possibly limiting the discriminative ability. Additionally, the physical fitness levels may have already been low across their lifespan, which has been confirmed in other studies with younger individuals with intellectual disabilities (Golubovic et al., 2012; Hilgenkamp et al., 2012b; Lahtinen et al., 2007; Oppewal et al., 2013; Salaun & Berthouze‐Aranda, 2012). The age‐related decline in physical fitness may therefore be less pronounced in people with intellectual disabilities, influencing the relationship with survival. The presented C‐statistics in this study, representing the prognostic value of the models, were also at the lower end of the range of those seen in other studies in the general population (0.66–0.82 for gait speed; Studenski et al., 2011). Because of the already low physical fitness levels in this study sample at baseline, it would be interesting to repeat this study including younger participants with possibly higher physical fitness levels. Including a broader spectrum of physical fitness levels may result in a better accuracy to discriminate between those who survived and those who died. Another explanation for the low accuracy is that it is quite unlikely that physical fitness, while adjusting for the personal characteristics, is the only factor influencing survival. Other health parameters, such as diseases and chronic conditions, but also lifestyle, socioeconomic and psychosocial environmental factors also influence survival (Kuh et al., 2009). However, we showed that a broad range of physical fitness components are predictive for survival, and therefore, one's physical fitness can act as an important indicator of one's health and to identify those who may benefit from training to improve their physical fitness and thereby reduce their mortality risk.

Auditive reaction time, muscular endurance and flexibility were not predictive for survival. Muscular endurance, as measured with chair rises, has been found to be predictive for survival in the general population (Cooper et al., 2010). This difference might be explained by the fact that studies in the general population had a follow‐up between six and 10 years. Therefore, a longer follow‐up might be needed to study the relationship between muscular endurance and survival in more detail. Previously, we did find muscular endurance to be predictive for a decline in daily functioning over a 3‐year follow‐up period in older adults with intellectual disabilities, and it can therefore be considered to be an important aspect for healthy ageing in people with intellectual disabilities (Oppewal et al., 2014, 2015), but maybe not for survival. Flexibility and auditive reaction time were also not predictive for a decline in daily functioning in older adults with intellectual disabilities (Oppewal et al., 2014, 2015).

In addition to being predictive for survival, we previously demonstrated that physical fitness was predictive for a decline in daily functioning, operationalized as basic and instrumental activities of daily living and mobility (Oppewal et al., 2014, 2015). Together these results emphasize the importance of being physically fit for an increased lifespan, with less dependency in those additional years. Physical fitness is not just a specific marker for the separate components such as strength, balance and cardiorespiratory fitness that represents the ability to carry out daily tasks (American College of Sports Medicine, 2018), but may also be considered a marker of one's general health status. The support and care system for people with intellectual disabilities must focus on keeping people with intellectual disabilities physically active and fit. Not just stimulating physical activity, but specifically improving physical fitness must become a basic part of the care and support of adults with intellectual disabilities. We know this is easier said than done, and this requires a behavioural change in both people with intellectual disabilities and the care system and environment around them. Behaviour change techniques are important for this, but not often used in lifestyle interventions for people with intellectual disabilities (Willems, Hilgenkamp, Havik, Waninge, & Melville, 2017). Additionally, barriers to become physically active should be targeted and all parties involved must cooperate and take responsibility for this to be successful (Bossink, van der Putten, & Vlaskamp, 2017). Enhancing a physical active lifestyle requires a change from a medical care perspective to a more preventive care perspective, with potentially saving long‐term healthcare costs.

Strong aspects of this study are the long follow‐up, the large sample size and the extensive physical fitness assessment at baseline. It is also the first study to assess the relationship between physical fitness and survival in older adults with intellectual disabilities. However, this study also had some limitations. Although the HA‐ID study has a near‐representative study population (Hilgenkamp et al., 2011), result may not be representative for the entire population of older adults with intellectual disabilities because of selection bias. First, adults without any form of registered care or support were not included in the HA‐ID study, and adults who only visit a day care centre or only receive ambulatory care were underrepresented. Second, adults with severe or profound intellectual disabilities and wheelchair users were underrepresented in the physical fitness measurements. Finally, 56 participants were lost to follow‐up. These participants were younger and had a better manual dexterity, comfortable walking speed and cardiorespiratory fitness. This deregistration could have been selective and related to the time of death. This needs to be taken into account while interpreting the results.

Further, besides looking at all‐cause mortality, it is also interesting to assess the relationship between physical fitness and cause‐specific mortality. In a previous study, we saw that diseases of the respiratory system, neoplasms and diseases of the circulatory system were the most common primary causes of death (Oppewal et al., 2018). It can be expected that certain causes of death are more stronger related to low physical fitness levels than others, for example, diseases of the respiratory and circulatory system (Elbaz et al., 2013; Oppewal et al., 2018). Insight into the relationship between physical fitness and cause‐specific mortality and morbidity, also taking into account medication use for these specific conditions, may help in understanding the underlying pathways linking physical fitness to survival. A longer follow‐up is needed to obtain sufficient power for these analyses.

In this study, we assessed the predictive value of physical fitness at a single time point. For more in‐depth analyses of the relationship between physical fitness and survival, and the influence of the rate and amount of decline in physical fitness over time, physical fitness measurements need to be performed as well after a follow‐up period, combined with survival data. This will allow for investigating the relationship between changes in physical fitness over time and survival. A large decline in physical fitness may be a better predictor for mortality than a measure at a single point in time, because individual physical fitness levels may change over time due to alterations in physical activity habits and other lifestyle and health factors. Improvements in physical fitness over time may increase survival chances, and large declines may lower survival (Lee et al., 2010). A recommendation for future research is to focus on the patterns of physical fitness in time, and the relationship between the direction and magnitude changes in physical fitness with survival. This information is also important for goal setting in training interventions.

In the general population, it is seen that associations are weaker in studies with longer follow‐up and including younger ages (Cooper et al., 2010). Because people with intellectual disabilities already have low physical fitness levels at younger ages than the general population (Hilgenkamp et al., 2012b; Lahtinen et al., 2007), it would be interesting to see whether the associations are different in a younger population with intellectual disabilities. The level of physical fitness achieved during young adulthood is important for physical fitness later in life, as well as the rate of decline (Dwyer et al., 2009; Malina, 2001). This stresses the importance of being fit already at a young age and makes it interesting to also study the relationship of physical fitness levels with survival at younger ages.

In conclusion, physical fitness was predictive for survival in older adults with intellectual disabilities. This stresses the need for older adults with intellectual disabilities to stay physically active and fit, and this has to become an important part of the care and support for older adults with intellectual disabilities.

## CONFLICT OF INTEREST

All authors declare no conflicts of interest.
